# Resolving 500 nm axial separation by multi-slice X-ray ptychography

**DOI:** 10.1107/S2053273318017229

**Published:** 2019-02-12

**Authors:** Xiaojing Huang, Hanfei Yan, Yan He, Mingyuan Ge, Hande Öztürk, Yao-Lung L. Fang, Sungsoo Ha, Meifeng Lin, Ming Lu, Evgeny Nazaretski, Ian K. Robinson, Yong S. Chu

**Affiliations:** a National Synchrotron Light Source II, Brookhaven National Laboratory, Upton, New York 11973, USA; b Shanghai Synchrotron Radiation Facility, Shanghai Institute of Applied Physics, Shanghai 201204, China; c Department of Mechanical Engineering, Ozyegin University, Istanbul 34794, Turkey; d Computational Science Initiative, Brookhaven National Laboratory, Upton, New York 11973, USA; e Center for Functional Nanomaterials, Brookhaven National Laboratory, Upton, New York 11973, USA; f Condensed Matter Physics and Materials Department, Brookhaven National Laboratory, Upton, New York 11973, USA; g London Centre for Nanotechnology, University College London, London WC1H 0AH, UK

**Keywords:** X-ray ptychography, multi-slice approach, nanostructures

## Abstract

Multi-slice X-ray ptychography offers an approach to achieve images with a nanometre-scale resolution from samples with thicknesses larger than the depth of field of the imaging system by modeling a thick sample as a set of thin slices and accounting for the wavefront propagation effects within the specimen. Here, we present an experimental demonstration that resolves two layers of nanostructures separated by 500 nm along the axial direction, with sub-10 nm and sub-20 nm resolutions on two layers, respectively. Fluorescence maps are simultaneously measured in the multi-modality imaging scheme to assist in decoupling the mixture of low-spatial-frequency features across different slices. The enhanced axial sectioning capability using correlative signals obtained from multi-modality measurements demonstrates the great potential of the multi-slice ptychography method for investigating specimens with extended dimensions in 3D with high resolution.

## Abstract 2

Combining multi-slice ptychography with multi-modality scanning probe microscopy reconstructs two planes of nanostructures separated by 500 nm with sub-20 nm lateral resolution, assisted by simultaneously measured fluorescence maps for decoupling low-spatial-frequency features.

## 1. Introduction   

Diffraction-based imaging techniques such as coherent diffraction imaging (Miao *et al.*, 1999 ▸) and its scanning variant ptychography (Rodenburg *et al.*, 2007 ▸) are being pursued to achieve the diffraction-limited spatial resolution beyond the focus size provided by X-ray optics. With low numerical aperture (NA) imaging systems, the achievable resolution is primarily limited by the X-ray wavelength and the maximum diffraction angle (Sayre *et al.*, 1998 ▸). For achieving higher resolutions with high-NA imaging systems, the thickness effect starts to make an impact, since the corresponding depth of field decreases dramatically with the enlarged NA as λ/NA^2^. As a result, the sample thickness is required to be thinner than the depth of field in order to obtain diffraction-limited resolution, δ*r*, defined by the Rayleigh criterion as 0.61λ/NA. Otherwise, the wavefront propagation effect becomes non-negligible inside the sample, and the interaction with the illumination cannot be modeled by a simple multiplicative operation, which makes the projection approximation invalid. The thickness limitation *T* has been analyzed to satisfy *T* ≤ *A*(δ*r*)^2^/λ, where the scaling factor *A* varies from 0.5 to 4.88 in different theoretical estimations (Rodenburg & Bates, 1992 ▸; Chapman *et al.*, 2006 ▸; Thibault *et al.*, 2008 ▸; Jacobsen, 2018 ▸) and a recent empirical observation suggests *A* = 5.2 (Holler *et al.*, 2014 ▸; Tsai *et al.*, 2016 ▸).

The multi-slice ptychography method (Maiden *et al.*, 2012 ▸) was proposed to overcome the thickness limitation by modeling a thick sample as a consecutive series of thin axial slices (Cowley & Moodie, 1957 ▸). Here, each slice satisfies the projection approximation, and the interaction of each slice with the incident beam can be computed by multiplying the projected sample’s transmission function within the slice (the object) by the illumination function (the probe). The obtained wavefront exiting one slice freely propagates through the separation distance and serves as the new probe function for the next slice. This process is repeated over all the slices within the sample. The wavefront exiting the last slice carries the depth information of the entire sample and propagates to the detector plane. The recorded far-field diffraction pattern is used to update the wavefront exiting from the last slice, where the probe and the object functions are decoupled using conventional ptychographic algorithms (Maiden & Rodenburg, 2009 ▸; Maiden *et al.*, 2017 ▸). The obtained probe function is then sequentially back-propagated to the previous slices, and updates the corresponding probe and object functions on each slice. After the initial demonstration with visible light, this method was soon implemented and gained accumulating interest in the X-ray regime (Suzuki *et al.*, 2014 ▸; Shimomura *et al.*, 2015 ▸; Tsai *et al.*, 2016 ▸).

The axial-sectioning capability of the multi-slice ptychography method enables a new approach to obtaining depth information for 3D imaging without sample rotation, unlike the conventional tomography method (Godden *et al.*, 2014 ▸). However, considering that the depth resolution δ*r*_*z*_ = 1.22λ/(NA)^2^ (Born & Wolf, 1999 ▸) is much poorer than the resolution δ*r* in the lateral plane by a factor of NA/2, and the residual low-frequency features sweep through reconstructed slices, using the multi-slice ptychography method alone does not produce clean and isotropic 3D information. Instead, combining multi-slice ptychography with tomography integrates complementary benefits, which not only extends the allowable sample thickness (Maiden *et al.*, 2012 ▸; Tsai *et al.*, 2016 ▸), but also is able to reduce the number of tomographic projections (Hovden *et al.*, 2014 ▸; Li & Maiden, 2018 ▸). Recent advances in scanning probe microscopy promote the integration of these two powerful techniques by alleviating the challenges of mixing features and improving the achievable depth resolution. Ptychography measurements carried out in the multi-modality mode (Deng *et al.*, 2015*b* ▸; Jones *et al.*, 2016 ▸; Silva *et al.*, 2017 ▸; Yan *et al.*, 2018 ▸; Deng *et al.*, 2018 ▸) collect correlative images with various contrast mechanisms simultaneously and provide an opportunity to better decouple the mixture of residual features in the multi-slice ptychography reconstruction. Since the blended low-spatial-frequency information can potentially be extracted from other imaging channels, such as X-ray fluorescence maps, this method can be used to separate the features on different slices. Improving the depth resolution of the multi-slice ptychography method plays a critical role in relaxing the Crowther criterion on the number of projections required for a proper tomography reconstruction (Tsai *et al.*, 2017 ▸; Li & Maiden, 2018 ▸; Jacobsen, 2018 ▸; Shimomura *et al.*, 2018*a* ▸). A highly convergent X-ray beam focused by multilayer Laue lenses (MLLs) (Huang *et al.*, 2013 ▸) has proven successful in facilitating the axial sectioning capability due to the large NA and fast-changing wavefront along the beam-propagation direction. In our previous study, we distinguished two layers of nanoparticles separated by 10 µm with sub-10 nm lateral resolution with 50 ms dwell time using MLLs (Ozturk *et al.*, 2018 ▸). Recently, a 1.4 µm-depth resolution was reported using the precession multi-slice ptychography approach with datasets collected at nine tilted angles (Shimomura *et al.*, 2018*b* ▸). Here, we report our work on multi-slice X-ray ptychography with the aim of pushing the depth resolution down to the sub-micrometre regime using a single 2D scan. Leveraging the axial resolving power provided by MLLs and the information extracted from the fluorescence imaging channel of the multi-modality measurement, two layers of nanostructures separated by 500 nm were successfully reconstructed with sub-10 nm and sub-20 nm lateral resolutions, respectively. This proof-of-principle demonstration shows the potential and benefit of employing correlative images from the multi-modality measurements to enhance the depth resolution of the multi-slice ptychography method.

## 2. Experimental results and discussion   

**Figure 1:**
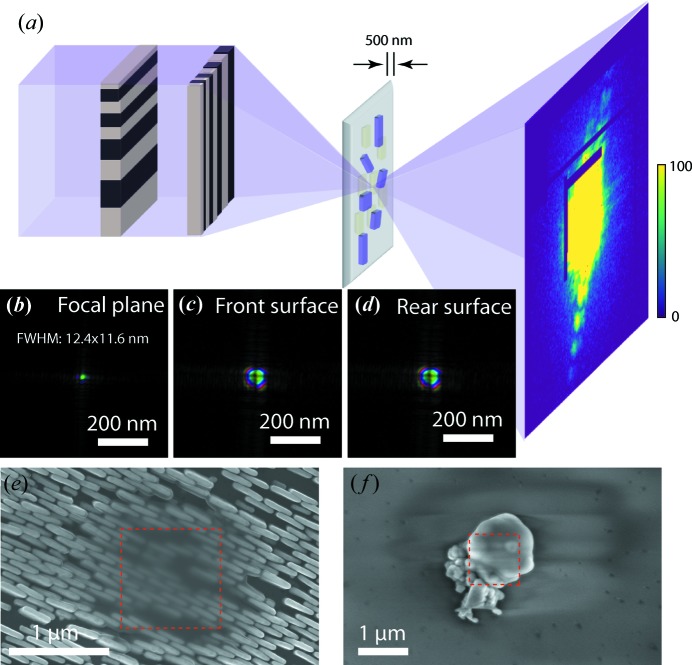
(*a*) Experimental setup for the multi-slice ptychography measurement. The order-sorting aperture (OSA) is not shown for simplicity. (*b*) The incident 12 keV X-ray beam was focused to a 12 × 12 nm spot by a crossed pair of MLLs. (*c*), (*d*) The X-ray wavefronts on the front and rear surfaces of the sample, which was placed 10 µm downstream from the focal plane. (*e*), (*f*) The SEM images of the gold zone plate structure and nickel oxide particles on the front and rear surfaces measured with 30 and 5 keV electron-beam energies, respectively. The dotted red boxes indicate the X-ray scanned area.

The multi-slice X-ray ptychography measurement was carried out at the Hard X-ray Nanoprobe beamline, National Synchrotron Light Source II (Chu *et al.*, 2015 ▸). The schematic of the MLL-based scanning probe microscope setup (Nazaretski *et al.*, 2015 ▸, 2017 ▸) with an off-axis focusing geometry (Yan & Chu, 2013 ▸) is illustrated in Fig. 1 ▸(*a*). The coherent portion of the incident 12 keV X-ray beam was selected by a 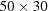 µm secondary source aperture. The filtered illumination propagated 15 m downstream and was focused by a crossed pair of MLLs with a 4 nm outermost zone width. A 53 × 43 µm aperture size covering 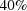 of a full MLL aperture delivered ∼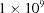 photons s^−1^ into a 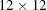 nm spot (Yan *et al.*, 2018 ▸). The sample was a zone plate structure fabricated with ∼450 nm-thick gold on a 500 nm-thick silicon nitride membrane. Nickel oxide particles were drop-casted on the rear surface of the membrane. The nickel oxide particles overlapped with the zone plate pattern on the front surface and formed a layered structure with 500 nm separation. Before the multi-slice ptychography measurement, a single-slice ptychography dataset was collected at a sample area with only the gold zone plate feature inside the field of view. The reconstructed wavefront was propagated to locate the focal plane, which is shown in Fig. 1 ▸(*b*). The sample was then moved to a location with both the gold zone plate feature and nickel oxide particles. The sample was also translated along the axial direction, and the front surface was 10 µm downstream from the focal plane to improve the overlapping condition with an enlarged beam (Bunk *et al.*, 2008 ▸; Huang *et al.*, 2017 ▸). The probe functions at the front and rear surfaces of the sample are shown in Figs. 1 ▸(*c*) and 1 ▸(*d*). Considering the depth of focus of the used MLLs is 3.9 µm, the divergence of the X-ray propagation over the sample thickness is negligible. After the X-ray measurement, the scanned area was surveyed with scanning electron microscopy (SEM). Figs. 1 ▸(*e*) and 1 ▸(*f*) are SEM images of the zone plate structure and nickel oxide particles. A noticeable pile of carbon accumulated over the X-ray scanned area on both surfaces. A 30 keV electron beam was used to penetrate the carbon layer to observe the gold feature underneath.

X-ray fluorescence (XRF) imaging was performed simultaneously to ensure both the gold zone plate feature and nickel oxide particles were captured inside the field of view. The multi-slice ptychography scan followed a Fermat spiral trajectory (Huang *et al.*, 2014 ▸) covering a 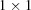 µm area with a radial step size of 25 nm. The diffraction patterns at 1414 scan positions were collected by a Merlin pixel array detector (Plackett *et al.*, 2013 ▸) with 55 µm pixels placed 0.35 m from the sample. A dwell time of 5 s was used for each data frame. Since the scan overhead is relatively small compared with the dwell time, the measurement was conducted using a step scan instead of the on-the-fly scheme (Pelz *et al.*, 2014 ▸; Deng *et al.*, 2015*a* ▸; Huang *et al.*, 2015*a* ▸), which would otherwise add more illumination modes in the calculation and thus significantly increase the computation burden in the reconstruction process. A typical diffraction pattern is shown in Fig. 1 ▸(*a*) with the maximum display range set to 100 photons. The diffraction intensity along the flares extends to the edge of the cropped 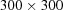 pixel array. This cropped array gives an effective NA of 0.024. The corresponding reconstruction pixel size Δ*r* and the expected depth resolution δ*r_z_* are about 2.2 and 226 nm, respectively.

**Figure 2:**
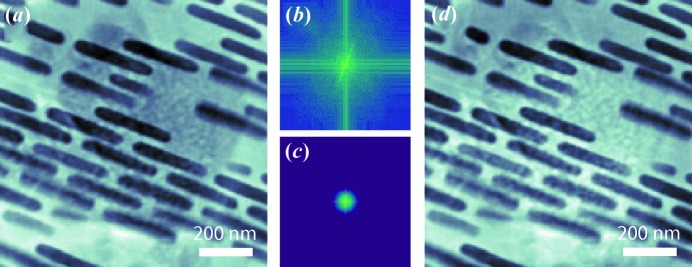
(*a*) Phase image from the single-slice ptychography reconstruction. (*b*) Fourier transform of the single-slice reconstruction image. (*c*) The nickel *K* map was interpolated and upsampled to match the dimension of the reconstructed image. The obtained fluorescence map [shown in Fig. 3 ▸(*d*)] is then Fourier transformed to reciprocal space. A low-pass filter was applied to select the low-spatial-frequency signal. (*d*) Normalized (*c*) is removed from (*b*) and Fourier transformed back to real space giving a modified phase image with the nickel oxide features removed.

**Figure 3:**
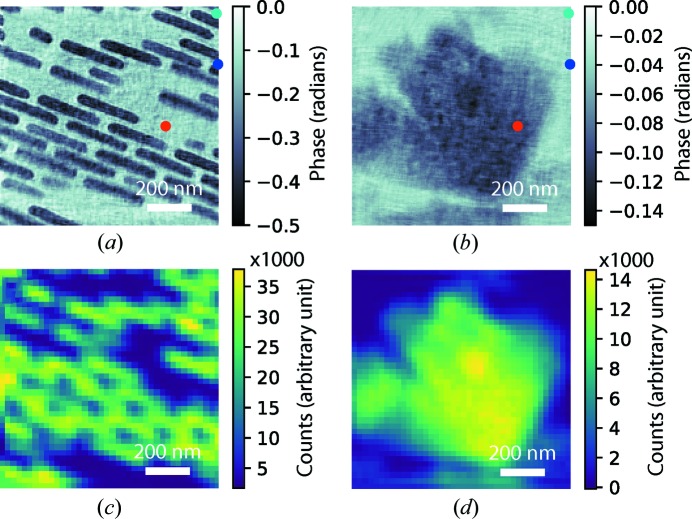
The phase images from the multi-slice ptychography reconstruction on the front (*a*) and the rear (*b*) surfaces. The cyan, blue and red dots indicate the scan positions for the background, the gold zone plate structure and the nickel oxide particles, respectively. (*c*), (*d*) The simultaneously measured Au *L* and Ni *K* maps interpolated from the 1414-point Fermat spiral scan pattern onto 40 × 40 mesh grids.

The dataset was first reconstructed using 1000 iterations of the difference map algorithm (Thibault *et al.*, 2008 ▸), assuming there was only one layer in the sample. The obtained phase image is shown in Fig. 2 ▸(*a*). The gold zone plate structure overlays with the rhombic shaped nickel oxide particles as expected in the single-slice reconstruction. It has been pointed out that the multi-slice ptychography reconstruction engine has difficulty distinguishing low-spatial-frequency signals, since these signals do not change over a long propagation distance (Tsai *et al.*, 2016 ▸). The mixture of low-spatial-frequency signal leaves ‘ghost’ residual features on the wrong planes. Since our experiment was conducted in a multi-modality scheme by collecting the far-field diffraction pattern and the fluorescence spectrum simultaneously, the low-spatial-frequency information representing the shape and size of the structures can be obtained from the XRF maps and can be used to separate the residual features. To verify this idea, we interpolated the gold *L* and nickel *K* fluorescence measurements from the 1414-point Fermat spiral pattern onto a 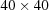 mesh grid. The obtained XRF maps are shown in Figs. 3 ▸(*c*) and 3 ▸(*d*), respectively. To remove the nickel oxide feature from the single-slice reconstruction, the interpolated nickel *K* map was upsampled to match the reconstruction image size, and then Fourier transformed to reciprocal space. A low-pass filter was applied to extract the low-spatial-frequency signal, as shown in Fig. 2 ▸(*c*). This selected signal was normalized and then subtracted from the Fourier transform of the single-slice reconstruction [shown in Fig. 2 ▸(*b*)]. The resulting pattern was inverse Fourier transformed back to real space and gave an image with the nickel oxide feature effectively removed, as shown in Fig. 2 ▸(*d*). The obtained image was used as the initial guess of the object image on the front slice for the subsequent multi-slice ptychography reconstruction. We found that the same procedure does not work well on removing the gold zone plate features. Since the gold zone plate structure consists of standing ‘bricks’ with various lengths and different spacings, the information about their shape, size and relative positions is encoded in more complicated signals in reciprocal space. Using only the low-spatial-frequency signal therefore cannot sufficiently represent the gold zone plate structure. Instead, we estimated the ranges of the phase and absorption contrasts introduced by the nickel oxide particle from the single-slice reconstruction, and used the nickel *K* map to estimate the shape, size and internal variations. Using this information, we generated the initial guess of the object image on the rear slice. The initial object image on the front surface can be generated using the gold *L* map in the same way, but we found that removing the ‘ghost feature’ from the single-slice reconstruction resulted in better recovered image quality. This observation suggests that the high-spatial-frequency signal provided by the single-slice reconstruction also plays a critical role in generating a better initial guess of the object slices for a cleaner axial separation.

The multi-slice ptychography reconstruction was conducted using 1000 iterations of the regularized ptychographic iterative engine (Maiden *et al.*, 2017 ▸). The probe function propagated from the wavefront reconstructed from the single-slice dataset was used as the starting guess for the probe on the front slice. The object images on two slices were fixed for the first 10 iterations to accelerate the initial probe refinement. After 10 iterations, both the object and probe images were updated in each iteration. The outputs of the last 200 iterations were averaged to obtain the final images. The reconstructed phase images for the gold zone plate structure on the front surface and the nickel oxide particles on the rear surface are shown in Figs. 3 ▸(*a*) and 3 ▸(*b*). As the features were better separated in the initial guesses, the residual mixture of the low-spatial-frequency features was significantly suppressed. The reconstructed images agree with the interpolated fluorescence maps very well.

**Figure 4:**
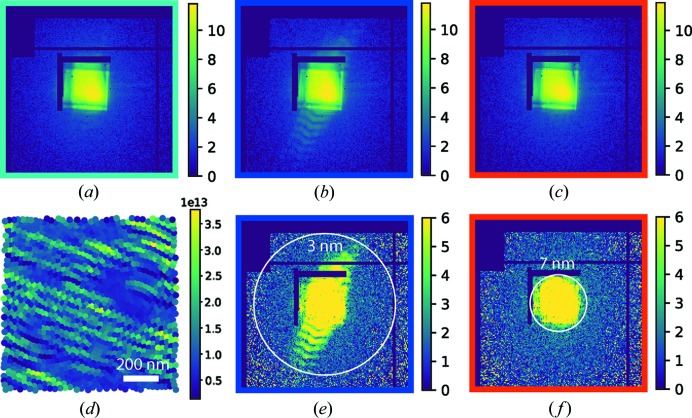
Far-field diffraction patterns displayed on a logarithmic scale at locations for (*a*) the background, (*b*) the gold zone plate structure on the front surface and (*c*) the nickel oxide particles on the rear surface. (*d*) The map of the number of scattered photons from the sample mainly represents the gold zone plate structure, which confirms that the gold structure scatters more strongly than the nickel oxide particles. (*e*), (*f*) The signal distribution extends to spatial frequencies corresponding to 3 and 7 nm at the scan positions on the gold zone plate structure and the nickel oxide particles, respectively.

**Figure 5:**
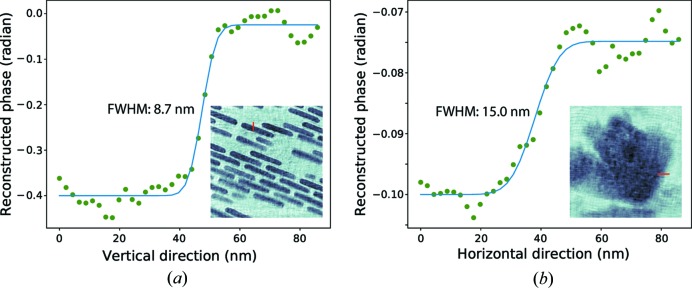
The error-function fittings to the edge profiles of the reconstructed phase images estimate 8.7 and 15.0 nm resolutions on (*a*) the front and (*b*) rear surfaces, respectively.

It can be seen that the apparent sharpness of the image on the front surface is much better than that on the rear surface. The gold zone plate structure produced an ∼0.5 radian phase contrast, while the nickel oxide particles produced an ∼0.15 radian phase contrast, which implies that the gold structure scatters 12 keV photons more strongly than the nickel oxide particles. The number of photons scattered by the sample on each scan position can be estimated to quantify the localized scattering intensity. Given that *I*(*q*) and *I*_0_(*q*) are the scattering patterns at a location inside the sample and in the background without the sample, the usable signal distribution above the expected Poisson noise level can be calculated (Dierolf *et al.*, 2010 ▸) as Ξ(*q*) = [*I*(*q*) − *I*_0_(*q*)]^2^/*I*_0_(*q*), and the number of scattered photons *N*_s_ can be obtained using *N*_s_ = ½[Σ_*q*_Ξ(*q*) − *N*_pix_], where *N*_pix_ is the number of pixels in the scattering pattern. We picked up three representative scan locations on the background, the gold zone plate structure and the nickel oxide particles. These locations are illustrated by the cyan, blue and red dots, respectively, in Figs. 3 ▸(*a*) and 3 ▸(*b*). The corresponding data frames are displayed in Figs. 4 ▸(*a*), 4 ▸(*b*) and 4 ▸(*c*) on a logarithmic scale. The number of scattered photons for each scan position is illustrated in Fig. 4 ▸(*d*), which mainly represents the gold zone plate structure on the front surface. The calculated signal distribution functions Ξ(*q*) for the gold zone plate structure and the nickel oxide particles are shown in Figs. 4 ▸(*e*) and 4 ▸(*f*), respectively. We can see that the gold zone plate structure scattered photons to the edge of the cropped array, while the signal distribution from the nickel oxide particles remained inside the numerical aperture region generated by the MLLs. The signal distribution analysis estimates the upper limits of the achievable resolutions to be 3 and 7 nm for the front and rear surfaces, respectively. The reconstruction resolutions were estimated by fitting an error function to the edge profiles on recovered images on two slices. Fig. 5 ▸ shows that the obtained resolutions are 8.7 and 15.0 nm on the front and rear surfaces, respectively.

Further improvements to the lateral and depth resolutions require collecting the diffraction signal at higher spatial frequencies and effectively decoupling low-spatial-frequency features. The first component can be achieved by using X-ray optics with high NA and high focusing efficiency, employing sophisticated data collection schemes such as the precession method, or increasing the exposure time. The second component can be fulfilled by utilizing the abundant information from the multi-modality measurements or from the multi-angular tomography measurements.

In our sample system, different elements are ideally separated on different slices, so the fluorescence maps work effectively on alleviating the residual low-spatial-frequency features. For general sample systems where different elements are not separated in the beam propagation direction, the decoupling of low-spatial-frequency features would rely on choosing the proper imaging mechanisms available in the multi-modality measurement scheme that best represents the structural properties of the sample. For example, the small-angle-scattering signal can be used for distinguishing materials with similar texture and electron density (Liebi *et al.*, 2015 ▸; Zhu *et al.*, 2018 ▸), and the Bragg diffraction can select crystal grains with different orientations (Huang *et al.*, 2015*b* ▸; Yau *et al.*, 2017 ▸). Meanwhile, recent algorithm development shows that combining multi-slice ptychography with tomography and exploiting the redundant measurements at different angles helps the decoupling of low-spatial-frequency features. This idea was first demonstrated by Li & Maiden (2018 ▸). Another recent development of the optimization algorithm shows a similar capability to obtain high-resolution 3D reconstructions with extended depth of field (Gilles *et al.*, 2018 ▸).

## 3. Conclusions   

We present our work on multi-slice X-ray ptychography, resolving two slices of nanostructures axially separated by 500 nm, with sub-10 nm and sub-20 nm lateral resolutions from a single 2D scan with 5 s dwell time per point. The highly convergent X-ray beam focused by large-NA MLLs provides a fast-changing wavefront, which facilitates the depth resolution enhancement. The high focusing efficiency of MLLs, which delivers ∼1 × 10^9^ photons s^−1^ onto the focal spot, is beneficial for collecting a high-spatial-frequency signal to further improve depth resolution. The synergy between multi-slice ptychography and multi-modality scanning probe microscopy offers opportunities to utilize information obtained from other imaging channels in order to assist in the isolation of low-spatial-frequency features. In this work, we demonstrated the use of simultaneously measured fluorescence maps to decouple two separated slices with different elemental compositions. We showed that having additional information or constraints on the sample can aid the depth resolution of the multi-slice ptychography method. The generalization of this approach in practical sample systems would rely on choosing adequate imaging channels to properly represent the structural properties of the specimen. With the potential of improving depth resolution in the multi-modality measurement scheme, multi-slice X-ray ptychography as a technique is expected to be a powerful tool for high-resolution 3D imaging of specimens with extended dimensions.
